# Geodetic Constraints on Segment-Scale Slip Rates and Interseismic Coupling Along the Havran–Balıkesir Fault Zone, NW Anatolia, Türkiye

**DOI:** 10.3390/s26082539

**Published:** 2026-04-20

**Authors:** İbrahim Tiryakioğlu, Halil İbrahim Solak, Ali Özkan, Cemil Gezgin, Eda Esma Eyübagil, Ece Bengünaz Çakanşimşek Ünlükaya, Kayhan Aladoğan, Çağlar Özkaymak, Mehmet Ali Uğur, Hasan Hakan Yavaşoğlu, Cemal Özer Yiğit, Bahadır Aktuğ, Vahap Engin Gülal

**Affiliations:** 1 Department of Geomatics Engineering, Afyon Kocatepe University, 03200 Afyonkarahisar, Türkiye; itiryakioglu@aku.edu.tr (İ.T.); maliugur@aku.edu.tr (M.A.U.); 2 Earthquake Implementation and Research Center of Afyon Kocatepe University, 03200 Afyonkarahisar, Türkiye; hisolak@aku.edu.tr (H.İ.S.); caglarozkaymak@aku.edu.tr (Ç.Ö.); 3 Distance Education Vocational School, Afyon Kocatepe University, 03200 Afyonkarahisar, Türkiye; 4 Osmaniye Vocational School, Osmaniye Korkut Ata University, 80000 Osmaniye, Türkiye; aliozkan@osmaniye.edu.tr; 5 Department of Geomatics Engineering, Aksaray University, 68100 Aksaray, Türkiye; cemilgezgin@aksaray.edu.tr; 6 Graduate School of Natural and Applied Sciences, Afyon Kocatepe University, 03200 Afyonkarahisar, Türkiye; edaeyubagil@gmail.com (E.E.E.); ece-bengunaz.cakansimsek@usr.aku.edu.tr (E.B.Ç.Ü.); 7 Osmancık Ömer Derindere Vocational School, Hitit University, 19030 Çorum, Türkiye; kayhanaladogan@hitit.edu.tr; 8 Department of Geological Engineering, Afyon Kocatepe University, 03200 Afyonkarahisar, Türkiye; 9 Department of Geomatics Engineering, Istanbul Technical University, 34100 Istanbul, Türkiye; yavasoglu@itu.edu.tr; 10 Department of Geomatics Engineering, Gebze Technical University, 41400 Gebze, Türkiye; cyigit@gtu.edu.tr; 11 Department of Geophysical Engineering, Ankara University, 06830 Ankara, Türkiye; aktug@ankara.edu.tr; 12 Department of Computer Engineering, Atlas University, 34408 İstanbul, Türkiye

**Keywords:** Havran–Balıkesir fault zone, GNSS, block modeling, interseismic coupling, slip partitioning, seismic hazard

## Abstract

**Highlights:**

**What are the main findings?**

- High spatiotemporal resolution GNSS velocity field derived from 77 sites reveals distinct along-strike kinematic heterogeneity along the Havran–Balıkesir Fault Zone.
- Segment-resolved block modeling identifies strong slip partitioning and high slip-deficit rates on the Turplu and Gökçeyazı segments.

**What are the implications of the main findings?**

- The Gökçeyazı segment exhibits significant slip-deficit rates (~4–6 mm/yr) and prolonged seismic quiescence, indicating a high potential for a future large earthquake.
- Segment-scale geodetic observations improve the understanding of interseismic coupling variations and seismic hazard along complex fault systems in western Anatolia.

**Abstract:**

This study presents a new high-resolution GNSS-derived velocity field and the first internally consistent, segment-resolved block model for the Havran–Balıkesir Fault Zone (HBFZ) in western Anatolia. Inversion of the GNSS velocity field was performed using a dense network of 77 sites within a 3D elastic half-space framework to estimate fault slip rates and interseismic coupling. The results reveal that the HBFZ behaves as a kinematically heterogeneous fault system, with deformation systematically partitioned along strike. Block-modeling results indicate pronounced along-strike variations in interseismic coupling and slip-deficit accumulation. While the westernmost Havran segment is weakly coupled and accommodates limited accumulation, the Turplu and Gökçeyazı segments emerge as major strain-accumulation zones with high and laterally continuous slip-deficit rates. In particular, the Gökçeyazı segment exhibits slip-deficit rates of ~4–6 mm/yr and nearly two millennia of seismic quiescence, implying the potential for a future large-magnitude earthquake (Mw ~7.1–7.3). The strong agreement between GNSS-derived deformation patterns and independent geological and paleoseismological constraints suggests that this segment is currently in an advanced stage of the seismic cycle. These findings highlight the importance of segment-scale geodetic observations for seismic hazard assessment in northwestern Anatolia.

## 1. Introduction

**Figure 1:**
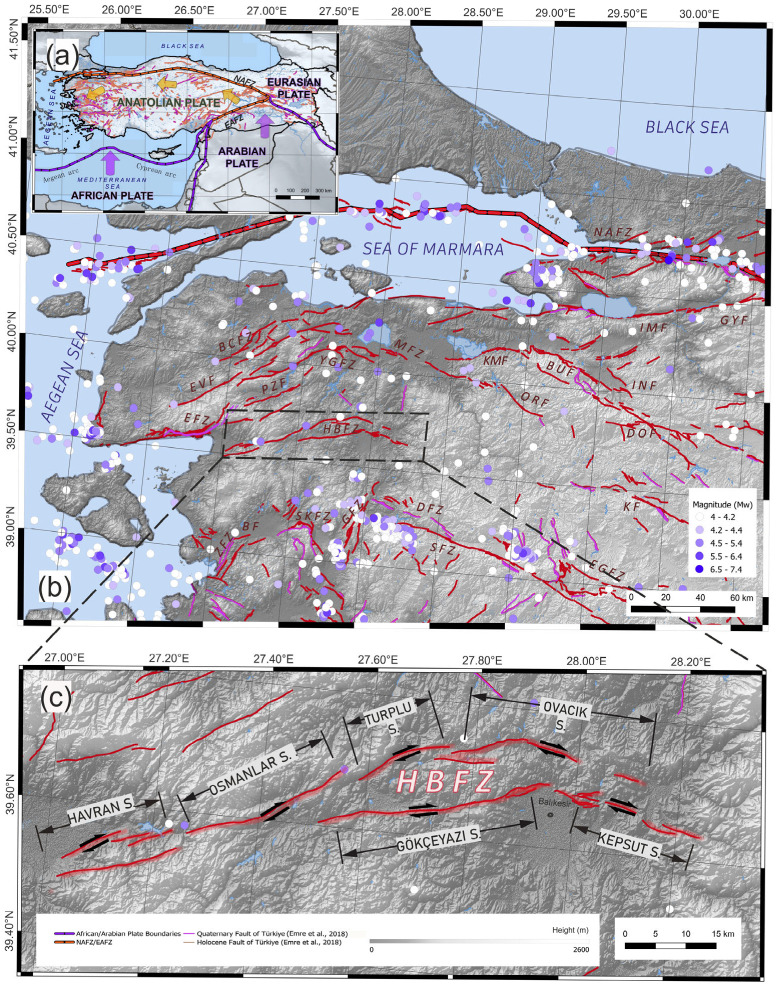
(**a**) Tectonic framework of Anatolian plate showing the relative motions of the African, Arabian, and Eurasian plates and the major fault systems (**b**) Active fault distribution and earthquake epicenters (Mw ≥ 4) in western Anatolia (**c**) Detailed map of the Havran–Balıkesir Fault Zone (HBFZ) illustrating its main segments (Fault taken from [15], Abbreviations: Bergama Fault: BF, Biga Çan Fault Zone: BCFZ, Bursa Fault: BUF, Edremit Fault Zone: EFZ, Emet–Gediz Fault Zone: EGFZ, Evciler Fault: EVF, Dodurga Fault: DF, Düvertepe Fauşt Zone: DFZ, Geyve Fault: GYF, Gelenbe Fault Zone: GFZ, Havran–Balıkesir Fault Zone: HBFZ, İnegöl Fault Zone: INF, Manyas Fault Zone: MFZ, M. Kemal Paşa Fault: KMF, North Anatolian Fault Zone: NAFZ, Orhaneli Fault, İznik Mekece Fault: IMF, Pazarköy Fault: PZF, Simav Fault Zone: SFZ, Soma Kırkağaç Fault Zone: SKFZ Yenice Gönen Fault Zone: YGFZ, Zeytindağ Fault Zone: ZFZ).

The neotectonic framework of the Anatolian Plate is primarily governed by the westward escape of the Anatolian block along the right-lateral North Anatolian Fault Zone (NAFZ) and the left-lateral East Anatolian Fault Zone (EAFZ), driven by the collision between the Arabian and Eurasian plates [1,2,3,4,5,6,7]. In northwest Anatolia, the NAFZ bifurcates into northern, central, and southern branches upon entering the Marmara region, with the transtensional character becoming more pronounced toward the south—moving from the transpression—dominated NAFZ toward the West Anatolia Extensional Province (WAEP). The southern branch, which accommodates a significant portion of the regional deformation, is represented by numerous parallel or sub-parallel dextral strike-slip faults extending between Lake İznik and the Aegean Sea [2,8,9,10,11]. One of these major structures, the Havran Balıkesir Fault Zone (HBFZ) (Figure 1), plays a critical role in the seismic hazard and risk assessments of the southern Marmara region and WAEP [12,13]. This zone, approximately 120 km in length, consists of the Havran-Balya (northern strand) and Balıkesir faults (southern strand) and is characterized by long-term slip rates ranging from 3.59 to 3.78 mm/yr, respectively [13]. The Havran-Balya Fault lies in the north, exhibits an en-echelon geometry with the Havran, Osmanlar, Turplu, and Ovacık segments, aligned from west to east, whereas the Balıkesir Fault is divided into the Gökçeyazı and Kepsut segments in the south [9,12,14,15,16] (Figure 1). Kinematic analyses indicate that the western and central segments of the Balıkesir Fault are characterized by strike-slip motion with a contractional component, whereas the easternmost Kepsut segment is dominated by normal/oblique-slip [12].

Given the tectonic complexity of the region, the geological framework of the HBFZ plays a key role in controlling its structural elements and deformation patterns. The HBFZ truncates the complex geological basement of NW Anatolia, which primarily comprises the Permian–Middle Triassic Karakaya Complex of the Sakarya Zone, the Late Cretaceous–Paleocene rocks of the İzmir-Ankara Zone, and the Oligocene Hallaçlar volcanics [12,13,17,18,19]. This basement is unconformably overlain by Early–Middle Miocene volcano-sedimentary sequences [12,20,21]. During the Pliocene and Quaternary, terrestrial clastics, fluvial sediments, and active alluvial fans accumulated within modern basins controlled by the fault zone [12,13]. The HBFZ has shaped the present-day morphology by tectonizing the contacts between these lithological units [12]. Particularly around Balıkesir and Gökçeyazı, Plio-Quaternary terrestrial units outcrop unconformably over the basement and Miocene volcanics [12,13]. These units are directly truncated by HBFZ segments, forming thick sequences along fault scarps. The presence of basal blocky levels, especially along the margins of the Balıkesir Basin, indicates high-energy sedimentation triggered by active faulting [9,12,13]. Both main strands of the HBFZ (Havran-Balya and Balıkesir) exhibit distinct segment boundaries and right-stepping en-echelon geometries. Geomorphic markers such as right-laterally deflected streams, shutter ridges, and fault-parallel elongated ridges are prominent along the fault traces. While the Turplu and Ovacık segments are separated by pull-apart basin geometries [13], the Kepsut segment displays more pronounced fault scarps, indicating a significant normal-slip component that enhances topographic relief [12]. Kinematic investigations by [12] reveal a three-stage late Cenozoic deformation history for the HBFZ. Inversion of fault-slip data demonstrates a temporal rotation of the principal stress axes. Phase 1 (pre-Pliocene) is characterized by left-lateral strike-slip faulting with a reverse component. Phase 2 (Plio-Quaternary) reflects the initiation of the North Anatolian Fault Zone (NAFZ) in the Southern Marmara region, dominated by right-lateral strike-slip motion under N-S contraction and E-W extension. The youngest stage, Phase 3 (Quaternary), is attributed to a transpressional regime involving N-S contraction and E-W extension. While several studies in the Southern Marmara report NW-SE trending contraction associated with right-lateral and reverse faulting [22,23,24], an extensional regime prevails further west along the northern margin of the Edremit Gulf [13,25]. This extension is interpreted as the result of mechanical interaction between the NAFZ and the Aegean Extensional System. Analyzing seismological data through moment tensor solutions in the Southern Marmara region, Yalçın et al. [26] found that earthquakes are primarily associated with NE-striking right-lateral strike-slip faults, alongside some E-W striking dip-slip normal faults. This integrated geological and tectonic framework is directly reflected in the spatial distribution of seismicity and deformation patterns observed along the HBFZ.

Western Anatolia, one of the most seismically active regions of the Anatolian plate and characterized by frequent swarm-type seismicity and high spatial earthquake probability [27], hosts the multi-segmented HBFZ. Although no large earthquakes (M > 6) have been recorded yet along the HBFZ during the instrumental period, historical earthquake catalogs document three events with intensity between VI and VIII (1577, 1896 and 1898) in and around Balıkesir [28]. Instrumental seismicity indicates that the HBFZ stands out with relatively limited earthquake activity despite being located within the highly seismically active western Anatolia. The recorded events are predominantly concentrated along the western and south part of the fault zone, particularly on the Havran, Osmanlar and Gökçeyazı segments. In contrast, the eastern segments exhibit a comparatively quieter seismic pattern, with notably fewer instrumental earthquakes observed toward the Ovacık and Kepsut segments [13,28]. This spatial variation in seismicity may suggest differences in stress release along the fault system, with some segments potentially accommodating deformation through longer interseismic periods. Despite this relatively limited instrumental seismicity, previous geological and paleoseismological studies indicate that several segments of the fault zone are capable of producing large earthquakes [13,15]. In this context, Sözbilir et al. [13] reported that the Gökçeyazı, Kepsut, and Ovacık segments have the potential to generate destructive earthquakes with moment magnitudes of up to Mw 7.2. Although the average recurrence interval for major late Holocene events is 1000–2000 years, the lack of surface-rupturing activity on the Gökçeyazı segment for over 2000 years underscores its status as a significant seismic gap [13]. The proximity of the fault zone to densely populated areas, including the Balıkesir city center—one of the major urban centers in the region hosting nearly 1.5 million inhabitants—underscores the substantial risk that accumulated tectonic strain poses to public safety. Understanding the present-day deformation characteristics of the fault zone is therefore critical for evaluating its seismic potential. Previous geodetic studies conducted in this tectonically active region [29,30,31] have primarily focused on large-scale deformation patterns in western Anatolia and the Marmara region, providing regional velocity fields and plate kinematic models. However, these studies do not resolve deformation at the scale of individual fault segments and therefore no prior geodetic research has focused specifically on the HBFZ and its individual fault segments. Consequently, the segment-scale strain accumulation pattern and present-day slip partitioning along the HBFZ remain poorly constrained.

Despite the geological evidence indicating that the HBFZ is an active structure and southern segments identified as a significant seismic gap with the potential to generate large earthquakes, the present-day geodetic characteristics of its individual segments remain largely unexplored. In particular, high-spatial-resolution geodetic observations capable of resolving segment-scale strain accumulation and short-term slip rates are essential for improving seismic hazard assessments in the region. In this study, we use a dense GNSS network to quantify present-day deformation, slip rates, and strain distribution along the individual segments of the HBFZ. By providing the first segment-scale geodetic constraints for this fault system, the results offer new insights into the current kinematics and slip partitioning of the fault zone, contributing to a better understanding of earthquake potential within this complex tectonic transition between the southern Marmara region and the WAEP.

## 2. Geodetic Characterization of the HBFZ and Its Surroundings

Although the HBFZ has been investigated through geological and structural analyses, including mapping and long-term slip-rate estimates [13], its present-day kinematics have not been resolved geodetically at the scale of its individual mapped segments. Regional GNSS investigations have substantially improved constraints on Anatolian plate motions and strain distribution within the broader Aegean–Anatolian domain [29,30,31,32,33,34]. However, GNSS networks used in prior studies were primarily designed to constrain large-scale block interactions rather than to resolve fault-specific kinematics along the HBFZ. Consequently, deformation associated with the HBFZ has largely remained embedded within regional block solutions, limiting independent evaluation of along-strike segmentation and interseismic slip behavior.

This limitation is particularly evident in early velocity-field solutions for the Marmara region [32] and in the continental-scale block model of [29], which provided robust constraints on regional deformation but included only sparse station coverage near the HBFZ. Within these frameworks, the fault zone was incorporated into large tectonic blocks, preventing resolution of along-strike variations in locking depth, slip partitioning, and segment-specific slip rates. Grid-based strain analyses by [30] improved regional spatial resolution; however, the temporal density of observations surrounding the HBFZ remained insufficient to resolve its internal segmentation. More recent dense-network studies conductued in western Anatolia [31] adopted smaller tectonic blocks and refined kinematic parameterizations within the İzmir–Balıkesir Transfer Zone. Despite these methodological advances, the HBFZ continued to be treated as a single structural entity within block modeling frameworks, and potential heterogeneities in interseismic coupling and strain accumulation along its mapped segments were not independently quantified. From a kinematic perspective, resolving segment-scale slip behavior requires observational coverage capable of separating rigid block motion from elastic strain accumulation localized along individual fault strands. In the absence of such resolution, slip rates inferred from regional models may represent spatial averages that obscure along-strike variations in locking state and seismic potential. Addressing this limitation requires a dense, segment-scale geodetic network and a kinematic framework capable of explicitly modeling individual fault boundaries [35,36,37].

## 3. GNSS Data and Methodology

Interseismic deformation and fault kinematics can only be robustly resolved when GNSS networks provide both adequate spatial density and appropriate geometric configuration relative to the fault system [38,39,40,41]. In particular, station density and along-strike distribution play a critical role in capturing segment-scale variations in locking depth, cosesimic deformation, slip partitioning and fault slip rates [41,42]. Sparse or irregularly distributed networks tend to smooth or obscure these variations, leading to oversimplified representations of fault behavior. Therefore, the design of a GNSS network must be explicitly tailored to the structural geometry and kinematic characteristics of the target fault zone in order to reliably resolve its heterogeneous deformation pattern [43].

**Figure 2:**
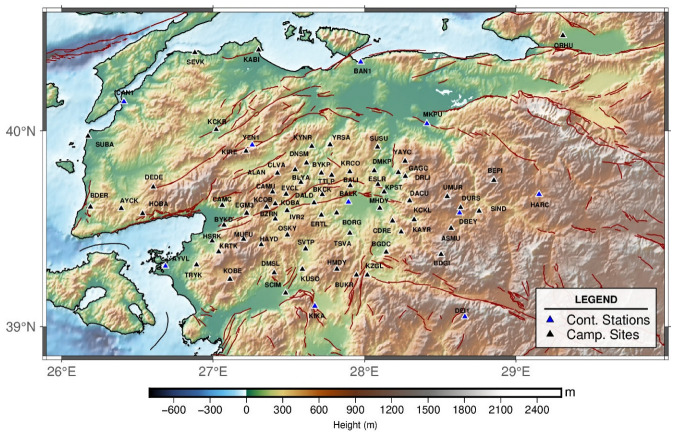
Spatial distribution of the GNSS network established to constrain the kinematics of the region. Continuous stations (blue triangles) and campaign sites (black triangles) are deployed across the active tectonic framework. Active faults are delineated by red lines compiled from [15].

A dedicated GNSS network, based on the region’s active tectonic framework and geological characteristics, was designed to robustly constrain the kinematics of the predominantly strike-slip HBFZ. The network geometry consists of profiles oriented as perpendicular as possible to the fault, with sites uniformly distributed on both sides of the fault zone to robustly resolve interseismic deformation. The initial configuration consisted of 64 campaign-style GNSS sites. In a subsequent phase, 13 continuously operating permanent GNSS stations were integrated into the network, resulting in a total of 77 sites. This hybrid configuration significantly improved temporal continuity and spatial resolution, ensuring dense coverage across the HBFZ while maintaining sufficiently broad spatial representation to characterize the regional deformation field surrounding the fault zone (Figure 2). To ensure long-term monument stability, permanent bronze benchmarks were installed at sites located on competent bedrock. Campaign measurements were performed using chain-mounted tripods, which minimize centering errors and improve repeatability between successive occupations. This measurement strategy was designed to ensure high positional accuracy and long-term stability of the geodetic observations. Based on this observation network the GNSS network provides continuous spatial coverage across all six mapped segments of the HBFZ. Within the kinematic inversion framework, each segment was explicitly implemented as an independent block boundary, enabling the estimation of segment-based geodetic slip rates. This segment-scale configuration ensures internally consistent short-term interseismic slip-rate estimates and allows direct comparison with geological constraints, thereby providing a refined basis for evaluating spatial variations in strain accumulation along the fault zone.

As part of this comprehensive geodetic network (Figure 2), four observation campaigns were carried out at 64 sites between 2022 and 2025. During each occupation, GNSS observations were acquired at a 30 s sampling interval for a minimum of two consecutive days, with at least eight hours of observation per day. This observation strategy ensured adequate data redundancy and temporal stability, allowing reliable estimation of interseismic site velocities. For several stations (see Table 1), GNSS datasets archived from previous geodetic investigations were incorporated, as these sites had been occupied during earlier campaigns. By integrating these earlier observations with the newly collected data, each campaign sites ultimately provided observations from at least three independent survey epochs spanning a minimum time interval of three years. This extended temporal coverage significantly enhances the robustness and reliability of the derived interseismic velocity field.

GNSS data were processed using the GAMIT/GLOBK software package v10.71 [44] following the strategy of [45], through a two-stage processing workflow. In the first stage, loosely constrained estimates of station coordinates, satellite orbital parameters, and Earth orientation parameters (EOPs), together with their full covariance matrices, were derived from double-difference carrier-phase observations. In the second stage, these loosely constrained, bias-fixed daily solutions and their associated covariance information were treated as quasi-observations and combined within the GLOBK framework. IGS final precise orbit and Earth rotation products were incorporated into the daily GAMIT solutions. Tropospheric delay was modeled using an “a priori model”, while zenith tropospheric delays were estimated as piecewise-linear parameters at 2 h intervals. Earth rotation parameters were adopted from the USNO_bull_b series. The daily solutions were subsequently combined using the GLOBK Kalman filter to generate continuous time series of site positions and velocities, which were aligned to the ITRF2014 reference frame [46]. The reference frame realization employed a 12-parameter Helmert transformation (three translations, three rotations, and their temporal rates), constrained to the ITRF2014 positions and velocities of 25 selected International GNSS Service (IGS) stations. Prior to velocity estimation, a stochastic noise model was implemented by introducing a random-walk process represented as a first-order Gauss–Markov (FOGMEX) process within the Kalman filtering framework [47]. This approach enables the simultaneous estimation of site velocities, offsets (e.g., equipment changes), seasonal signals, and more realistic velocity uncertainties. Following [47], the minimum random-walk noise level was set to 0.04 mm^2^/yr to avoid underestimation of uncertainties, ensuring that horizontal velocity uncertainties do not fall below approximately 0.1 mm/yr [48]. Reference frame stabilization was evaluated using the weighted root mean square (WRMS) of the horizontal residual velocities of the 25 selected IGS stations, yielding a value of approximately 0.13 mm/yr. The resulting Eurasia-fixed velocity field of the broader region surrounding the HBFZ is presented in Section 5. Finally, the time series of all continuous stations were carefully examined for transient deformation signals. No detectable coseismic or postseismic deformation signals associated with recent major earthquakes (2014-Çanakkale earthquake (Mw: 6.7) or 2023 Kahramanmaraş earthquakes (Mw:7.8, 7.7) were identified within the study region; therefore, no coseismic or postseismic corrections were applied to the GNSS time series [49,50].

## 4. Block Modeling

To characterize the block kinematics of Western Anatolia and the Northern Aegean, researchers have widely adopted rigid micro-block models, to explain the westward extrusion of the Anatolian plate and the Hellenic slab rollback drive regional extension [29,51,52]. In this context, deformation in the study region arises from a complex interplay of transtensional mechanisms and a prominent strike-slip shear zone that traverses the WAEP [12,13,14,53]. Collectively, these studies provide the tectonic and kinematic framework necessary for constructing the block model and defining the structural geometry of the HBFZ.

In this study, a block modeling approach was implemented to resolve the interseismic fault kinematics of the HBFZ by inverting GNSS velocities from both continuous stations and campaign sites. Using the TDEFNODE software (Version 2023.07.18) [54,55] within the framework of a 3D elastic homogeneous half-space based on the routines of [56], the block modeling approach facilitates the estimation of block rotation rates, fault slip rates, fault coupling ratios, and internal strain within the blocks [57,58]. In this approach, tectonic plates are defined as blocks (closed polygons) that are assumed to be quasi-rigid in their interior while rotating around specific Euler poles, and the model geometry is implemented by establishing a priori depth contours for the seismogenic zone.

The robustness of the TDEFNODE inversion in resolving complex fault interactions and interseismic strain partitioning has been recently demonstrated in various tectonic settings globally using TDEFNODE in the Sumatra subduction zone [59], the Alaska-Aleutian subduction zone [60], and northern Central America [61], as well as similar block modeling approaches in the Pamir region of Central Asia [62]. Collectively, these studies emphasize the critical role of dense geodetic arrays in capturing spatial variations in deformation, including along-strike coupling variations and depth-dependent locking patterns in subduction zones [59,60], along-strike variations in fault slip rates in intracontinental settings [62], and post-seismic afterslip distributions [61].

A major drawback of this approach occurs in the presence of sparse geodetic constraints. In such cases, fault slip rate estimations become highly dependent on and sensitive to the assumed block configurations and predefined locking depths. This limitation is commonly addressed by employing dense GNSS networks and applying checkerboard tests to assess whether the geodetic data sufficiently constrain fault kinematics [63,64,65]. In TDEFNODE, we use a block modeling approach in a homogeneous elastic half-space that neglects lateral and depth-dependent heterogeneities in elastic properties. This assumption is, however, widely adopted in interseismic block modeling studies [66,67,68] because: (1) first-order deformation patterns are primarily controlled by fault slip rates and locking distributions rather than second-order elastic heterogeneity, and (2) the lack of high-resolution 3D elastic structure at the scale of our study area prevents robust parameterization of heterogeneity. In fact, elastic properties vary laterally (e.g., due to sediment basins) and with depth (e.g., due to increasing confining pressure), and these heterogeneities could bias inferred slip rates and locking distributions. For example, lower rigidity in sedimentary basins would amplify elastic strain for a given slip deficit, potentially leading to overestimated locking if heterogeneity is ignored. Reference [69] demonstrated that heterogeneous models predict approximately 20% less slip than elastic half-space models in regions with deep slip and good geodetic coverage. Similarly, [70] showed that incorporating elastic heterogeneity shifts coupling estimates and reduces moment accumulation rates compared to homogeneous models. Based on these findings, we estimate that material heterogeneity could bias our fault slip rates by up to 20%, with the largest potential biases expected on fault segments adjacent to sedimentary basins where lower rigidity amplifies elastic strains. The along-strike pattern of interseismic coupling, however, is likely to remain robust because first-order deformation patterns are primarily controlled by fault slip rates and locking distributions rather than second-order elastic heterogeneity [66,67]. A quantitative assessment of these effects would require 3D finite-element modeling [69], which is beyond the scope of this study but is an important direction for future studies.

**Figure 3:**
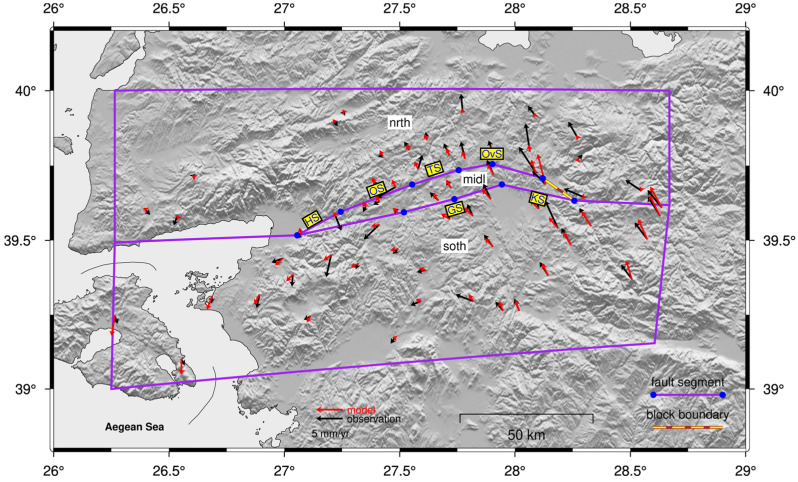
Proposed block model geometry for the study region. Blue circles correspond to nodes defined on the fault planes, connecting fault segments. The yellow-brown dashed line represents a block boundary with no corresponding fault segment and does not accumulate strain.

To derive slip rates and coupling ratios, we propose a block model consisting of three microblocks: the Northern block (reference block, “nrth”), the Middle block (“midl”), and the Southern block (“soth”). These blocks are delineated by the northern and southern strands of the fault zone in the study region. From west to east, the northern strand comprises the Havran Segment (HS), Osmanlar Segment (OS), Turplu Segment (TS), and Ovacık Segment (OvS), collectively known as the Havran-Balya Fault. Conversely, the southern strand is composed of the Gökçeyazı Segment (GS) and the Kepsut Segment (KS), which together form the Balıkesir Fault. In Figure 3, purple segments bounded by nodes represent fault segments that accumulate strain, whereas purple lines lacking end nodes act as non-straining block boundaries. Red and black vectors represent modeled and observed velocities, respectively, with 95% confidence ellipses. A 3-parameter rotation was applied to the observed velocities during the inversion to optimize the reference frame alignment.

Our model incorporates fault planes with sub-vertical dip angles between 87° and 89° [16]. The geometry of the HBFZ is defined by a nodal grid comprising 6 nodes along-strike for the northern strand and 5 for the southern strand, with both strands consisting of 8 nodes in the down-dip direction. We define slip rate as the GNSS-derived interseismic relative motion between adjacent blocks (i.e., the tectonic loading rate). Slip-deficit (φ), ranging from 0 to 1, represents the fraction of this slip rate not accommodated by aseismic creep, thus contributing to elastic strain accumulation. Fully locked faults (φ = 1) accumulate strain at the full slip rate, whereas fully creeping faults (φ = 0) accumulate no elastic strain. In our model, faults are fully locked (φ = 1) in the shallow zone (0–5 km). Between 5 km and the fully creeping depth, φ is estimated by inversion within the range 0–1, representing partial locking. Below this transition zone, the model assumes full creep (φ = 0). The slip distribution follows an exponential function [65]. Specifically, we adopt their justification that the exponential function reduces the overprediction of coastal velocities and the underprediction of inland velocities observed in linear downdip transition models. In our study region, the vast majority of GPS velocities are inland velocities, making the exponential function particularly appropriate because it avoids the systematic bias inherent to linear transition models. Additionally, another important reason for preferring the exponential function is that during the inversion adjustment, no prior constraints were applied on either the shape of the distribution or the boundaries of the fully locked and fully creeping zones. The exponential function allows a smooth, data-driven transition without imposing arbitrary discontinuities, which would otherwise require prior assumptions. Model parameters are derived by minimizing the residuals between observed and modeled velocities, providing a quantitative assessment of the model-data misfit. This misfit minimization is performed using the simulated annealing technique, which incorporates the reduced chi-squared (χ^2^) statistic along with regularization terms designed to penalize roughness in the slip distributions [39]. The methodological approach and algorithms implemented in this study follow the comprehensive framework described detailly in [64,71].

Model robustness is ensured through trade-off analysis, which identifies the optimal balance between data misfit and model roughness for the selection of smoothing coefficients. In our inversion, we employed a Laplacian smoothing approach to regularize the slip distribution on the fault patches. This method minimizes the second derivative of the slip with respect to the along-strike (x) and down-dip (ω) directions. The smoothing penalty function is defined as:

$$Penalty=\;A_{1}{\left(\frac{\partial^{2}S}{\partial x^{2}}\right)}^{2}+\;A_{2}\;{\left(\frac{\partial^{2}S}{\partial \omega^{2}}\right)}^{2}$$

where S represents the slip amplitude, and A_1_ and A_2_ are the scaling factors for the penalties in the along-strike and down-dip directions, respectively. To yield physically plausible slip distributions, the transition between distinct kinematic states is governed by these smoothing coefficients, which effectively control the gradient of coupling across the fault surface.

**Figure 4:**
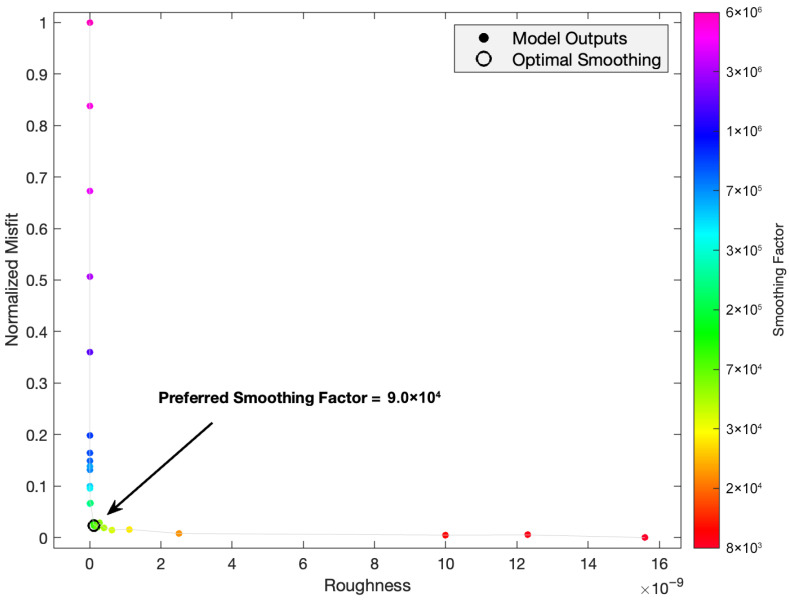
Normalized misfit versus model roughness of the regularization in the inversion (preferred smoothing coefficients are 9 × 10^4^ in along-strike and down-dip directions).

The smoothing coefficients were determined empirically by analyzing trade-off curves across a wide range of parameter values. Following the empirical approach widely used in block modeling, we utilized the L-curve criterion (Figure 4). The L-curve illustrates the trade-off between the data fit and smoothness of the solution. We identified the corner of this curve, which is the point of maximum curvature, as the optimal threshold where the data are fit sufficiently without over-parameterizing the model. Any further decrease in misfit beyond this point would require an unjustifiable increase in model roughness, indicating potential over-fitting. Based on this quantitative metric, the optimal smoothing coefficients for both fault strands were selected as 9 × 10^4^ (A_1_ and A_2_), ensuring a stable inversion that captures significant slip-deficit heterogeneity.

**Figure 5:**
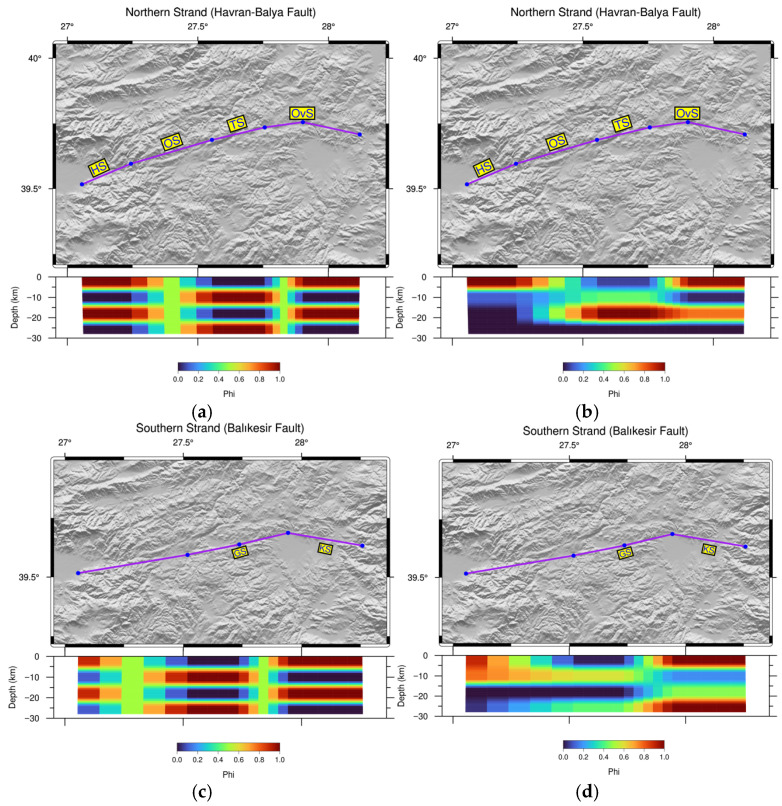
Checkerboard tests for slip distribution simulations along the northern strand ((**a**,**b**): HS, OS, TS, and OvS) and southern strand ((**c**,**d**): GS, KS) of the HBFZ. The left panel (**a**,**c**) corresponds to the forward model, displaying input slip patches at varying depths. The right panel (**b**,**d**) shows the corresponding slip distribution recovered by the model after checkerboard inversion.

Furthermore, the resolving power and sensitivity of the geodetic array are evaluated via synthetic checkerboard tests, assessing the model’s capacity to recover prescribed locking patterns across various depths. We implemented checkerboard tests using slip patches whose dimensions match the fault segments along both the northern and southern strands of the HBFZ (Figure 5). In this approach, uniformly locked fault patches are discretized using a 2 × 2 nodal mesh and assigned binary states representing either full coupling or free slip. The fault patches are discretized into small rectangular tiles to better approximate the curved fault geometry and to capture spatial variations in slip, since the [56] solution assumes uniform slip on rectangular dislocations. By projecting these patterns into a forward model, we derive synthetic velocities at the actual GPS site locations, incorporating stochastic noise to mimic real observations. To assess the ability of the inversion to recover the original locking pattern, the proposed model is subsequently solved using these perturbed velocities.

The checkerboard test results indicate that the spatial resolution of the model is strongly depth-dependent. At shallow depths (<10 km), the checkerboard patterns are recovered robustly across both strands, demonstrating that the network configuration is well suited to resolve first-order features of interseismic coupling along the entire fault zone. With increasing depth, however, the recovery gradually deteriorates due to the inherent loss of resolution and the decreased sensitivity of surface GNSS velocities to deeper slip variations.

Along the northern strand, the checkerboard recovery tests confirm that the entire seismogenic zone, spanning all segments from Havran to Ovacık, is adequately resolved (Figure 5a,b). The spatial extent and amplitude of the input slip-deficit anomalies are successfully reproduced, supporting the interpretation of heterogeneous coupling patterns and segment-scale locking behavior documented in this study. For the southern strand, the resolution tests further validate the robustness of the slip-deficit estimates, particularly across the central and eastern domains where the Gökçeyazı and Kepsut segments are located (Figure 5c,d). Accordingly, our network is also capable of recovering significant slip-deficit anomalies along these segments, supporting the interpretation that they constitute primary zones of strain accumulation. The checkerboard results thus confirm that the observed slip-deficit patterns reflect actual tectonic signals rather than artifacts of the inversion or network geometry.

## 5. Results

**Figure 6:**
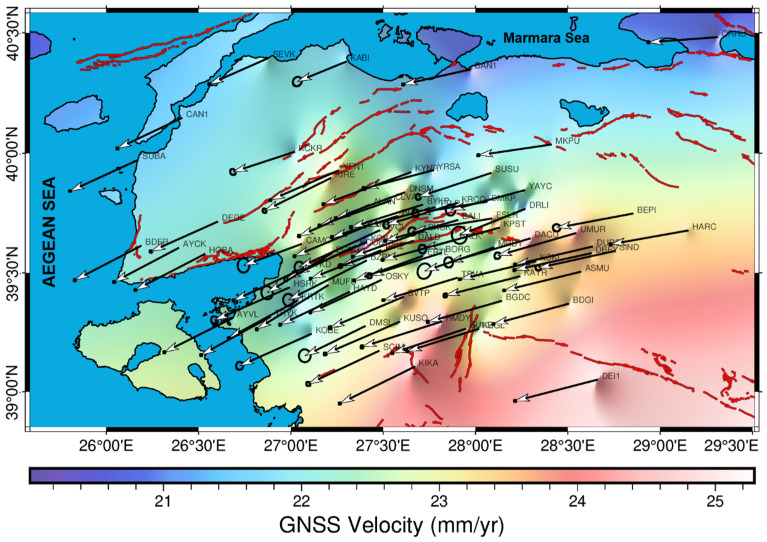
Contemporary velocity field of the study area (ellipses in 95% confidence, ITRF2014 Eurasia-fixed reference frame). Arrows indicate vectoral magnitudes of the GNSS sites.

In this study, we present a high-resolution GNSS-derived velocity field for the HBFZ and its surroundings and corresponding block modeling results that constrain segment-scale slip rates with locking behavior along the entire fault system. Regarding the 77-site network, GNSS velocities along the HBFZ reveal a systematic spatial pattern; east–west velocity components range from −15 to −18 mm/yr in the northern and western sectors, increasing to −23 to −26 mm/yr toward the eastern and, particularly, southeastern parts of the study area. The north–south velocity components reach up to −10 to −13 mm/yr in the southern sector and progressively decrease to −1 to −6 mm/yr toward the north (Figure 6). Taken together, these observations indicate that the velocity field along the HBFZ is characterized by a predominant west–southwest-directed motion, with velocity differences of up to ~5 mm/yr between the eastern–western and northern–southern ends of the fault zone. Examination of the Eurasia-fixed velocity field across the HBFZ reveals a systematic and spatially coherent deformation pattern. The study region is characterized predominantly by westward motion [29,31], with horizontal velocities increasing progressively toward the eastern, and particularly the southeastern sectors. Also, GNSS sites located along the southern strand of the HBFZ exhibit relatively higher velocities (~25 mm/yr), which gradually decrease northward across the fault system (~20 mm/yr).

**Figure 7:**
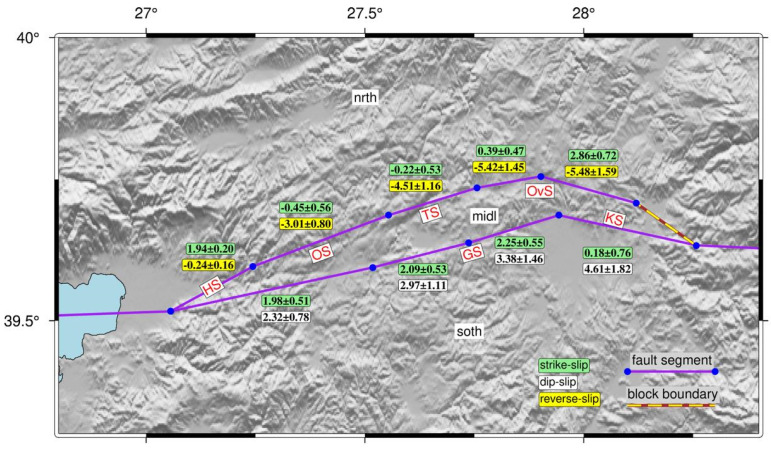
Estimated fault slip rates for the proposed block model. Green, white and yellow labels indicate strike-slip, dip-slip and reverse-slip rates, respectively. The model is referenced to the northernmost block (abbreviated as “nrth”). (Abbreviations: Havran Segment (HS), Osmanlar Segment (OS), Turplu Segment (TS), Ovacık Segment (OvS), Gökçeyazı Segment (GS), Kepsut Segment (KS)).

**Figure 8:**
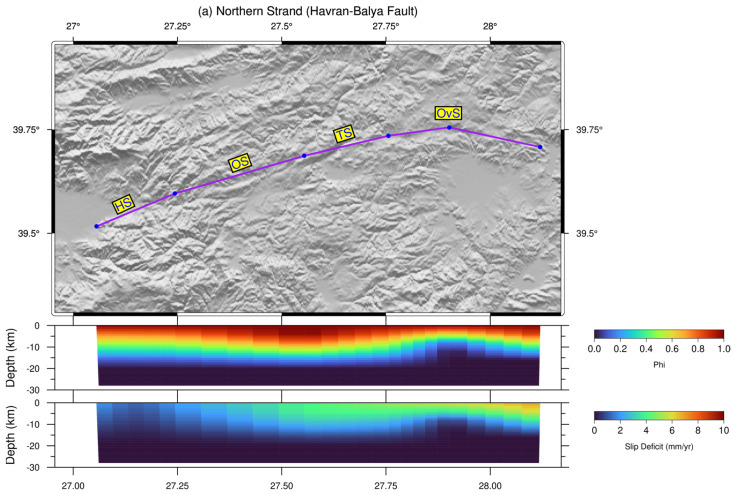

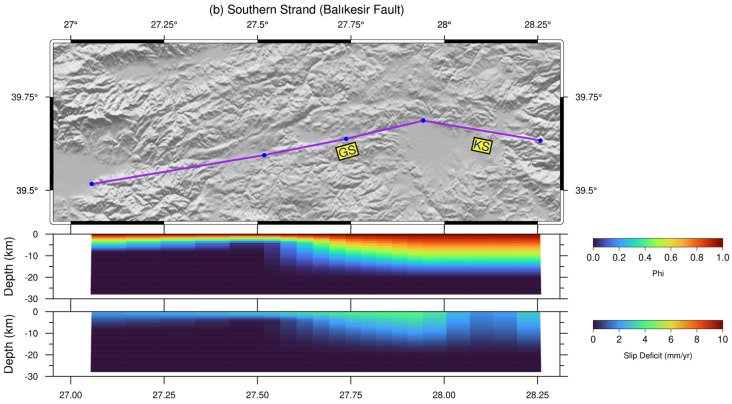
Distribution of coupling ratios and slip deficit rates along the northern strand (**a**) and southern strand (**b**) of the HBFZ. In the upper panel, the purple line represents the block boundary, and blue circles represent the nodes delineating fault segmentation. In the lower panel, colored cross-sections depict the estimated coupling ratios and slip deficit rates, respectively. (Abbreviations: Havran Segment (HS), Osmanlar Segment (OS), Turplu Segment (TS), Ovacık Segment (OvS), Gökçeyazı Segment (GS), Kepsut Segment (KS)).

These spatial gradients indicate a clear acceleration of velocity vectors both in magnitude and azimuth toward the east–southeast, whereas a distinct deceleration trend is observed toward the northern part of the network. Such kinematic behavior reflects along-strike variations in strain accumulation and suggests differential partitioning of deformation between the southern and northern strands of the HBFZ. Following the velocity field, segment-scale slip rates along the HBFZ and coupling ratios were estimated for the first time by inverting the GNSS velocities within an elastic half-space dislocation model (Figure 7 and Figure 8). The northern strand comprises four segments from west to east: Havran (HS), Osmanlar (OS), Turplu (TS), and Ovacık (OvS), whereas the southern strand defines as two segments: Gökçeyazı (GS) and Kepsut (KS).

At the western end of the northern strand, the HS is characterized by a right-lateral strike-slip rate of ±2 mm/yr with low 1σ uncertainties, indicating predominantly strike-slip behavior. In contrast, strike-slip rate along the OS is not statistically significant, but it accommodates ±3.1 mm/yr of reverse slip. Toward the eastern part of the northern strand, the TS and OvS exhibit dominant reverse slip ranging between ~4.5 and 5.5 mm/yr. While the strike-slip component along the TS is not statistically significant, the OvS displays a notable strike-slip component of approximately ~3 mm/yr. Along the southern strand, in contrast to the dominant normal faulting observed on the KS, the segments located further west exhibit oblique fault character with a combination of normal and strike-slip components.

Slip-deficit values are very low on both segments, 0–1 mm/yr on the HS and <2–4 mm/yr on the OS, throughout most of the seismogenic depth (Figure 8a). Strike-slip rate along the TS is also insignificant, similar to the OS. However, together with the OvS, the TS accommodate >5 mm/yr of reverse-slip, gradually increasing from west to east, indicating compression-dominated deformation along the northern strand. Slip deficit increases markedly along the TS, reaching ±4.5–5 mm/yr at shallow to mid-crustal depths (Figure 8b).

Along the Ovacık Segment (OvS), strike-slip rates vary between ±0.6 and 3.0 mm/yr, whereas the segment exhibits a reverse slip rate of >5 mm/yr, indicating that deformation along the OvS is largely controlled by compression. The OvS exhibits moderate slip-deficit values that increase gradually with depth, reaching ±4–6 mm/yr down to 10 km depth. Along the eastern part of the southern strand, the GS represents the only segment where both strike-slip and normal slip rates are statistically significant. The findings indicate that this ENE–WSW trending and north-dipping segment exhibits an oblique-slip normal character and controls the Quaternary basin on the hanging wall north of the Gökçeyazı settlement area from the south. The GS along the southern strand accommodates ±2 mm/yr of right-lateral strike-slip motion, accompanied by a substantial dip-slip rate of ±3.0–3.4 mm/yr. The GS displays the highest and most continuous slip deficit along the southern strand, attaining 4–6 mm/yr at shallow depths. The KS, also on the southern strand, exhibits statistically insignificant strike-slip motion, but a significant dip-slip rate of ±4.5 mm/yr, indicating dominantly extensional regime at the eastern end. The KS shows weak locking, with low slip deficit values at shallow depths. Our model inversion along the HBFZ reveals prominent heterogeneity because of changing slip rates between −5 and +2 mm/yr, and fault characteristics between the northern and southern strands.

## 6. Discussion

According to GNSS-derived velocity inversions, the fault slip deficit rates and coupling ratios reveal a heterogeneous interseismic deformation pattern along the HBFZ. Contrary to a uniformly locked strike-slip model, the fault zone displays distinct along-strike heterogeneity in coupling and faulting mechanism, implying that elastic strain accumulation is controlled by segment-scale structural geometry and the prevailing stress regime. Beyond segment-scale slip rates, the findings provide a broad-scale characterization of the kinematic regime along the northern and southern strands of the HBFZ. Overall, the northern strand is characterized by a deformation regime dominated by reverse faulting components along the Havran, Osmanlar, Turplu, and Ovacık segments. In particular, the very low strike-slip rates and distinct reverse slips observed along the Osmanlar–Turplu–Ovacık segments suggest that the northern strand is governed primarily by a transpressional tectonic regime. Within this tectonic setting, the HS exhibits more complex kinematic behavior, with dextral strike-slip motion and compression operating simultaneously, which renders it the most heterogeneous section of the northern strand [13], the only kinematic analysis study based on geological fault surface measurements on the HBFZ, indicates—similar to findings of this study—that the OS and OvS in particular are characterized by transpressional tectonic movements.

The southern strand accommodates clear slip partitioning: the GS exhibits both right-lateral strike-slip and dip-slip mechanisms, whereas the KS is dominated by pure normal faulting. This indicates a transtensional regime along the southern strand, with slip partitioned into oblique extension in the west and pure extension in the east. The pure normal faulting character of the KS was also reported in the geological investigations conducted by [12]. In the same study, both contraction-dominated deformation and transtensional deformation were identified along the GS, with this reactivation attributed to distinct tectonic phases. The findings obtained in this study indicate that a transtensional regime is currently active on this segment. When the segment-scale results obtained in this study are compared with the fault kinematics reported by [15], the right-lateral character inferred for the HS and OvS segments along the northern strand is consistent with the previously proposed fault character. However, in contrast to the right-lateral character suggested for the OS and TS segments, the results of this study indicate the presence of a significant reverse component. Along the southern strand, the slip characteristics obtained for the KS are in agreement with previous interpretations [9,15,72]. In contrast, although the GS was previously described as predominantly right-lateral, our results reveal that normal faulting appears to be the dominant component of deformation along this segment. Collectively, these findings demonstrate that the HBFZ behaves as a kinematically heterogeneous fault system rather than a single coherent structure. Deformation is strongly partitioned along the fault zone, with slip mechanisms and kinematic components varying markedly between individual segments. The geodetic findings may be compatible with the paleostress orientations proposed for the region [12], although such a comparison should be made cautiously due to the different timescales represented by paleostress indicators and present-day geodetic observations. The GNSS-based slip-deficit and coupling patterns further reveal strong along-strike contrasts between weakly coupled segments with limited strain accumulation and strongly locked segments characterized by higher slip-deficit values during the interseismic period.

Comparison of geological and geodetic slip rates along the HBFZ shows that the observed differences of approximately 1–1.5 mm/yr are broadly consistent once differences in observation timescales and measurement uncertainties are considered. Geological slip rates derived from paleoseismological trenches and offset geomorphic markers represent millennial-scale average deformation, whereas GNSS-derived slip rates reflect the present-day interseismic behavior of the fault system. Consequently, small segment-scale discrepancies between the two datasets are mechanically expected rather than contradictory [73,74,75].

Along the KS and OvS, paleotrench-based slip rates of 1–1.5 mm/yr [13] are compatible with the low-to-moderate geodetic slip rates. The presence of statistically significant dip-slip rates and moderate slip-deficit values indicates that surface deformation is partly suppressed and elastic strain is accumulating at seismogenic depths. In the GS, the paleoseismological slip rate of 1–1.5 mm/yr is in close agreement with the GNSS-derived slip rate of ±2 mm/yr, whereas the higher geomorphic slip rate (±3.59 mm/yr) likely reflects shorter-term surface processes and localized displacement. Similarly, in the TS, the high geomorphic slip rate (±3.78 mm/yr) is consistent with GNSS results showing dominant reverse motion and elevated slip-deficit values, implying efficient long-term strain accumulation despite relatively lower surface velocities. Overall, the limited differences between geological and geodetic fault slip rates along the HBFZ mainly reflect the time-dependent nature of fault slip within the earthquake cycle. Their combined use provides a more robust framework for interpreting segment-scale mechanical behavior and seismic potential. Against this background of overall consistency, the GNSS-derived coupling and slip-deficit patterns offer a basis for investigating how strain accumulation varies along individual segments of the HBFZ.

The westernmost HS exhibits very low slip deficit values and shallow locking, indicating weak coupling over most of the seismogenic depth range. This behaviour is consistent with geological observations showing reduced cumulative displacement toward the western end of the HBFZ, supporting the interpretation of the HS as a mechanically weak segment with limited long-term strain accumulation [13]. In contrast, OS displays transitional coupling behavior, characterized by moderate slip deficit values concentrated at mid-seismogenic depths. This pattern is typical of partially coupled fault segments located within step-over or restraining-bend geometries, where deformation is distributed between adjacent segments rather than localized on a single locked asperity [13,76]. Accordingly, the OS likely acts as a mechanical transition zone, facilitating strain transfer along the fault system. In comparison, the TS, located east of the OS, exhibits significantly higher slip-deficit rates and a wider locked zone, indicating that it is more efficient at storing elastic strain. This behavior is consistent with long-term geological slip-rate estimates derived from displaced geomorphic markers, implying sustained fault activity over Quaternary timescales [13]. Hence, the observed slip deficit rates reflect a full interseismic loading phase rather than a transient deformation. A broadly similar, though more heterogeneous, coupling pattern characterizes the OvS, where slip deficit rates reach maximum values near the eastern end of the zone. Paleoseismological findings indicate that repeated surface-rupturing earthquakes with recurrence periods of approximately 1000 years have occurred along this segment, suggesting that elastic strain is released episodically through large-magnitude events. Furthermore, the high slip deficit at the eastern edge of the OvS is consistent with fault-end effects, where strain concentration is mechanically expected. Together with its paleoseismic records, this segment represents a seismically mature and mechanically locked section of the northern strand, capable of generating large-magnitude earthquakes. At the eastern termination of the HBFZ, the KS exhibits distinct mechanical behavior, dominated by normal faulting under transtensional conditions. Despite its extensional character, the slip-deficit indicates moderate coupling and significant elastic strain accumulation. Similar observations have been reported for normal-fault-dominated segments interacting with strike-slip systems in western Anatolia, where extension does not necessarily preclude seismic locking [77,78]. Repeated large earthquakes identified in paleoseismological trench studies further support the high seismogenic potential of the KS. The GS represents the major strain accumulation zone along the southern strand of the HBFZ. It sustains high slip-deficit under transtensional forces, extending throughout most of the seismogenic layer and closely resembling the coupling patterns documented for recognized seismic gaps beneath the Sea of Marmara [64,79].

The block modeling approach based on GNSS velocities in this study provides quantitative estimates of fault slip rates and interseismic coupling ratios, enabling a robust assessment of seismic hazard along the HBFZ. Paleoseismological investigations indicate that the GS has not experienced a surface-rupturing earthquake for approximately 2000 years, implying a remarkably long period of seismic quiescence. When this prolonged interseismic period is considered together with the geodetically inferred long-term slip rate of ±1.8 mm/yr, the cumulative slip deficit on the GS is estimated to have reached ±3.6 m. Assuming coseismic release of this accumulated slip deficit is released coseismically as a characteristic event, empirical magnitude–displacement scaling relationships [80] suggest a corresponding large earthquake of Mw ±7.1–7.3. In their paleoseismological trenching study conducted [13], the maximum earthquake magnitude for the fault as Mw 7.2, applying the magnitude-fault length empirical relationships of [80]. This estimate represents an upper-bound scenario in which the majority of the accumulated strain is released during a single rupture. In a potential future earthquake, the coseismic displacement associated with the GS may vary depending on rupture extent, fault segmentation, and the degree of slip partitioning along the fault, particularly if the accumulated strain is released through partial ruptures or distributed across multiple segments. Nevertheless, the coexistence of high slip deficit, strong interseismic coupling, and nearly two millennia of seismic quiescence may indicate that the GS of the HBFZ is in a relatively advanced stage of the seismic cycle, although this inference remains uncertain due to its reliance on model-dependent parameters and indirect indicators. The GS segment therefore likely represents a significant source of regional seismic hazard in northwestern Western Anatolia.

Building on these findings, the results of this study provide a robust basis for decision-makers and risk assessment frameworks by identifying segment-scale variations in slip-deficit accumulation and interseismic coupling. In particular, the recognition of the Gökçeyazı segment as a zone of elevated seismic potential highlights its critical role in regional seismic hazard assessments. Such segment-scale geodetic constraints offer a fundamental framework for prioritizing high-risk zones and improving region-specific mitigation strategies. In this context, the identification of high slip-deficit segments provides essential input for urban planning, suggesting that building standards, construction practices, and infrastructure resilience should be re-evaluated in areas where elevated seismic hazard is anticipated. The integration of these findings into seismic risk mitigation strategies may significantly enhance preparedness and reduce vulnerability in densely populated regions such as Balıkesir and its surroundings.

In parallel, the use of a dense GNSS network in this study substantially improves the spatial resolution of the velocity field and enables the detection of along-strike variations in slip rates and interseismic coupling at the segment scale. Compared to sparser geodetic networks, which tend to produce spatially averaged deformation fields, the present approach allows the resolution of segment-specific kinematic behaviors that would otherwise be difficult to resolve. In particular, variations in fault character, slip components, and velocity gradients along individual segments can be captured with a dense network configuration, whereas such heterogeneities are commonly smoothed out in lower-resolution datasets [81,82]. This highlights that high-density geodetic observations are essential for accurately characterizing fault kinematics and for reliably identifying mechanically distinct segments within complex fault systems.

While the use of a dense GNSS network significantly improves the spatial resolution of fault kinematics, several data-related and methodological limitations should be considered. The accuracy and reliability of geodetic velocity estimates are influenced by factors such as observation time span, temporal distribution of measurements, and the precision of campaign-based GNSS observations. In particular, campaign measurements may introduce additional uncertainties compared to continuous stations due to temporal gaps in data acquisition. Nevertheless, it is well established that extending the observation time span and incorporating measurements from previous years can significantly improve the accuracy of velocity estimates, as longer time series better constrain long-term station motions [83]. In this study, the inclusion of multi-year observations contributes to the robustness of the derived velocity field, although some degree of uncertainty remains, particularly in segments with limited temporal coverage. These considerations indicate that, despite the overall reliability of the results, the presented estimates should be interpreted within the context of data availability and model assumptions; further improvements can be achieved through longer observation periods, an increased number of campaign measurements, and the expansion of continuous GNSS station coverage in future studies.

## 7. Conclusions

This study presents the first segment-scale investigation of contemporary deformation patterns along the HBFZ, utilizing a dense GNSS network and a unified block-modeling approach. By applying a consistent modeling strategy across all segments, we provide the first internally consistent geodetic slip-rate and locking model spanning the entire fault system, enabling a robust comparison of along-strike variations in slip partitioning and interseismic coupling. The findings demonstrate that the HBFZ does not behave as a kinematically homogeneous fault system but instead exhibits pronounced along-strike heterogeneity, with present-day deformation strongly controlled by segment-scale structural geometry and the regional stress regime.

The Havran Segment exhibits very low slip-deficit values and shallow locking, indicating a mechanically weak segment with limited long-term strain accumulation, whereas the Osmanlar Segment functions as a mechanical transition zone that facilitates strain transfer along the fault system. The Turplu and Ovacık Segments are characterized by high slip-deficit rates and broad locked zones, making them highly efficient at storing elastic strain. Among them, the Gökçeyazı and Kepsut Segments emerge as the most significant strain-accumulation zones, characterized by high slip-deficit rates and strong interseismic coupling. When combined with paleoseismological evidence of prolonged seismic quiescence, the geodetic results suggest that the Gökçeyazı Segment in particular has accumulated a substantial amount of elastic strain, indicating that it is in an advanced stage of the seismic cycle and constitutes a critical source of regional seismic hazard in northwestern Anatolia.

These findings highlight the importance of segment-scale geodetic observations for resolving spatial variations for improving realistic seismic hazard assessments in complex fault systems. However, these results must be interpreted considering several limitations. The elastic half-space assumption and the simplified fault geometry introduce trade-offs between slip rate and coupling, particularly at depth where resolution decreases. Differences between geodetic and geological slip rates likely reflect their respective positions within the earthquake cycle rather than fundamental inconsistencies. Despite these uncertainties, the strong agreement between GNSS-derived slip-deficit patterns and independent geological and paleoseismological observations supports the robustness of the segment-based interpretations presented here. A comprehensive understanding of fault behavior in all its aspects in such a complex tectonic setting also requires the integration of multiple datasets. Future studies should therefore focus on combining geodetic observations with seismological, geological, and paleoseismological data to better constrain fault geometry, slip behavior, and stress evolution. Multidisciplinary approaches integrating GNSS data with seismic monitoring, fault imaging, and stress modeling will help reduce model-dependent uncertainties and improve the reliability of seismic hazard assessments in regions such as the HBFZ. In particular, future work incorporating refined fault geometries, improved depth-dependent resolution, and additional geological constraints will further reduce parameter trade-offs and enhance quantitative estimates of seismic hazard along the HBFZ.

## Figures and Tables

**Table 1:** Information on GNSS stations used in this study, including site coordinates, observation type, observation period, and number of observations.

No	Site	Lon	Lat	Obs. Type	First-Last Obs	Obs. Number	No	Site	Lon	Lat	Obs. Type	First-Last Obs	Obs Number
1	ALAN	27.425	39.785	Camp.	2000.7–2020.7	5	40	HAYD	27.318	39.421	Camp.	2007.7–2024.5	3
2	ASMU	28.572	39.505	Camp.	2006.9–2024.9	4	41	HMDY	27.818	39.296	Camp.	2008.5–2024.9	3
3	AYCK	26.394	39.606	Cont.	2018.1–2025.0	Daily	42	HOBA	26.535	39.58	Camp.	2018.4–2024.9	3
4	AYVL	26.686	39.311	Cont.	2008.1–2025.0	Daily	43	HSRK	26.994	39.44	Camp.	2022.1–2024.9	4
5	BALI	27.906	39.722	Camp.	2013.3–2024.9	5	44	IVR2	27.489	39.595	Camp.	2006.9–2024.9	5
6	BALK	27.894	39.639	Cont.	2008.8–2019.8	Daily	45	KABI	27.301	40.41	Camp.	2016.5–2021.5	3
7	BAN1	27.975	40.349	Cont.	2014.5–2025.0	Daily	46	KAYR	28.242	39.486	Camp.	2006.9–2024.9	5
8	BDER	26.19	39.614	Camp.	2000.7–2021.5	4	47	KCKL	28.328	39.549	Camp.	2006.9–2024.9	4
9	BDGI	28.506	39.371	Camp.	2005.3–2024.5	5	48	KCKR	27.021	40.007	Camp.	2016.4–2022.6	4
10	BEPI	28.854	39.749	Camp.	2011.4–2024.5	4	49	KCOB	27.353	39.613	Camp.	2007.1–2024.9	7
11	BGDC	28.143	39.383	Camp.	2013.3–2024.5	5	50	KIKA	27.672	39.106	Cont.	2008.8–2025.0	Daily
12	BKCK	27.721	39.677	Camp.	2006.9–2024.9	5	51	KIRE	27.218	39.897	Camp.	2017.5–2021.5	3
13	BLYA	27.58	39.739	Camp.	2006.9–2024.9	5	52	KOBA	27.411	39.63	Camp.	2007.2–2024.5	3
14	BORG	27.816	39.583	Camp.	2008.8–2024.5	4	53	KOBE	27.112	39.244	Camp.	2011.5–2018.5	4
15	BUKR	27.947	39.265	Camp.	2007.1–2024.5	4	54	KPST	28.13	39.69	Camp.	2022.5–2024.9	5
16	BYKD	27.072	39.521	Camp.	2007.1–2022.7	3	55	KRCO	27.903	39.793	Camp.	2022.1–2024.9	5
17	BYKP	27.715	39.786	Camp.	2006.9–2024.5	4	56	KRTK	27.037	39.384	Camp.	2007.1–2024.9	5
18	BZRN	27.411	39.552	Camp.	2022.1–2024.9	4	57	KUSO	27.59	39.295	Camp.	2013.4–2024.5	5
19	CAMC	27.06	39.622	Camp.	2022.1–2024.9	4	58	KYNR	27.652	39.922	Camp.	2006.9–2024.9	4
20	CAMU	27.393	39.69	Camp.	2006.9–2024.9	5	59	KZGL	28.018	39.265	Camp.	2006.9–2024.5	4
21	CAN1	26.411	40.148	Cont.	2022.4–2025.0	Daily	60	MHDY	28.101	39.607	Camp.	2020.9–2024.9	4
22	CDRE	28.185	39.542	Camp.	2020.9–2024.5	4	61	MKPU	28.412	40.037	Cont.	2018.1–2025.0	Daily
23	CLVA	27.542	39.803	Cont.	2006.9–2024.9	4	62	MUFU	27.202	39.449	Camp.	2007.1–2024.9	4
24	DACU	28.298	39.645	Camp.	2022.4–2024.9	5	63	ORHU	29.31	40.482	Cont.	2018.1–2025.0	Daily
25	DALD	27.667	39.635	Camp.	2006.9–2024.5	3	64	OSKY	27.491	39.471	Camp.	2007.1–2024.9	4
26	DBEY	28.628	39.583	Cont.	2018.1–2025.0	Daily	65	SCIM	27.479	39.173	Camp.	2008.8–2024.5	4
27	DEDE	26.605	39.715	Camp.	2018.4–2022.6	3	66	SEVK	26.88	40.396	Camp.	2016.5–2022.6	6
28	DEI1	28.663	39.051	Cont.	2021.1–2025.0	Daily	67	SIND	28.757	39.592	Camp.	2007.4–2024.9	3
29	DMKP	28.064	39.798	Camp.	2022.1–2024.9	4	68	SUBA	26.174	39.973	Camp.	2016.5–2022.6	3
30	DMSL	27.404	39.277	Camp.	2006.1–2024.5	3	69	SUSU	28.086	39.918	Camp.	2016.4–2023.4	3
31	DNSM	27.617	39.837	Camp.	2006.9–2024.9	4	70	SVTP	27.611	39.4	Camp.	2008.8–2024.5	4
32	DRLI	28.27	39.769	Camp.	2022.4–2024.9		71	TRYK	26.891	39.317	Camp.	2007.1–2024.5	3
33	DURS	28.635	39.611	Camp.	2000.6–2024.6	8	72	TSVA	27.903	39.48	Camp.	2006.9–2024.9	5
34	EGM3	27.222	39.582	Camp.	2020.6–2024.5	3	73	TTLP	27.783	39.775	Camp.	2006.9–2024.4	4
35	ERTL	27.715	39.572	Camp.	2008.8–2024.9	6	74	UMUR	28.546	39.667	Camp.	2006.9–2022.5	3
36	ESLR	28.089	39.728	Camp.	2020.9–2024.9	4	75	YAYC	28.268	39.847	Camp.	2020.9–2024.5	4
37	EVCL	27.482	39.679	Camp.	2022.1–2024.9	4	76	YEN1	27.259	39.929	Cont.	2020.1–2025.0	Daily
38	GAGC	28.223	39.789	Camp.	2020.9–2024.9	4	77	YRSA	27.773	39.93	Camp.	2006.9–2024.9	3
39	HARC	29.153	39.678	Cont.	2008.9–2025.0	Daily							

## Data Availability

The data presented in this study are available on reasonable request from the first author. The data are not publicly available due to privacy and ethical restrictions.

## Abbreviations

The following abbreviations are used in this manuscript:

EOPs	Earth Orientation Parameters
FOGMEX	First-Order Gauss–Markov
GAMIT/GLOBK	GNSS Analysis at MIT/Global Kalman Filter
GNSS	Global Navigation Satellite System
GPS	Global Positioning System
IGS	International GNSS Service
ITRF2014	International Terrestrial Reference Frame 2014
TDEFNODE	TDEFNODE software
WRMS	Weighted Root Mean Square
